# Physiological and transcriptional mechanisms associated with cadmium stress tolerance in *Hibiscus syriacus* L.

**DOI:** 10.1186/s12870-023-04268-x

**Published:** 2023-05-29

**Authors:** Xiang Li, Lanlan Liu, Shixian Sun, Yanmei Li, Lu Jia, Shili Ye, Yanxuan Yu, Komivi Dossa, Yunpeng Luan

**Affiliations:** 1 The First Affiliated Hospital of Yunnan University of Traditional Chinese Medicine, Kunming, 650021 China; 2 Key Laboratory for Forest Resources Conservation and Utilization in the Southwest Mountains of China, Ministry of Education, Southwest Forestry University, Kunming, 650224 China; 3 Yunnan Key Laboratory of Plateau Wetland Conservation, Restoration and Ecological Services, Southwest Forestry University, Kunming, 650224 China; 4 Department of Life Technology Teaching and Research, School of Life Science, Southwest Forestry University, Kunming, 650224 China; 5 Faculty of Mathematics and Physics, Southwest Forestry University, Kunming, 650224 China; 6 CIRAD, UMR AGAP Institut, 34398 Montpellier, France

**Keywords:** *Hibiscus syriacus*, Cadmium, Transcriptome, Antioxidant system, Differentially expressed genes, Transcription factors

## Abstract

**Background:**

Cadmium (Cd) pollution of soils is a global concern because its accumulation in plants generates severe growth retardation and health problems. *Hibiscus syriacus* is an ornamental plant that can tolerate various abiotic stresses, including Cd stress. Therefore, it is proposed as a plant material in Cd-polluted areas. However, the molecular mechanisms of *H. syriacus* tolerance to Cd are not yet understood.

**Results:**

This study investigated the physiological and transcriptional response of “Hongxing”, a Cd^2+^-tolerant *H. syriacus* variety, grown on a substrate containing higher concentration of Cd (400 mg/kg). The Cd treatment induced only 28% of plant mortality, but a significant decrease in the chlorophyll content was observed. Malondialdehyde content and activity of the antioxidant enzymes catalase, peroxidase, and superoxide dismutase were significantly increased under Cd stress. Transcriptome analysis identified 29,921 differentially expressed genes (DEGs), including 16,729 down-regulated and 13,192 up-regulated genes, under Cd stress. Functional enrichment analyses assigned the DEGs mainly to plant hormone signal transduction, transport, nucleosome and DNA processes, mitogen-activated protein kinase signaling pathway, antioxidant process, fatty acid metabolism, and biosynthesis of secondary metabolites. Many MYB, EP2/ERF, NAC, WRKY family genes, and genes containing metal binding domains were up-regulated, implying that they are essential for the Cd-stress response in *H. syriacus*. The most induced genes were filtered out, providing valuable resources for future studies.

**Conclusions:**

Our findings provide insights into the molecular responses to Cd stress in *H. syriacus*. Moreover, this study offers comprehensive and important resources for future studies toward improving the plant Cd tolerance and its valorization in phytoremediation.

**Supplementary Information:**

The online version contains supplementary material available at 10.1186/s12870-023-04268-x.

## Background

The increasing pollution of the environment by unessential heavy metals, including Lead (Pb), Cd, Arsenic (As), Chromium (Cr), and Mercury (Hg), through expanding industrialization and disruption of natural biogeochemical cycles is a problem of global concerns [1]. Due to their nonbiodegradability, heavy metals accumulate in soils and cause severe environmental damage, affect plant growth and development, and pose health risks to humans [1, 2]. Among them, Cd is considered to be one of the most toxic, as its excessive accumulation in crops reduces their production, and causes serious human health problems via the food chain [3–5]. Cd is watersoluble and is therefore easily taken up from the soil by roots and transported to shoots and other organs [6]. Even in small amounts, its absorption negatively affects essential nutrients’ uptake and homeostasis in plants, leading to the disruption of metabolic processes [4]. The majority of agricultural soils have been contaminated with Cd through the utilization of sludge, phosphate fertilizers, pesticides, mining, irrigation water containing Cd, and urban traffic [5, 7]. Therefore, understanding the molecular mechanisms involved in Cd tolerance in plants will help to minimize Cd concentrations in plant tissues. Moreover, it will contribute to the development of novel Cd tolerant varieties and ultimately reduce human Cd intake.

Cd^2+^ induces toxicity in plants by inactivating biomolecules. Specifically, it substitutes some essential metal ions (Fe^2+^ and Zn^2+^) or obstructs functional groups; induces oxidative stress by stimulating the production of ROS (reactive oxygen species); and disrupts the activity of several enzymes (by binding to proteins with thiol radicals) [8]. These toxic mechanisms of Cd^2+^ accumulation cause severe damage to plants, including growth retardation; decrease in the content of chlorophyll, carotenoid, and water; reduction of leaf surface, biomass, yields, and photosynthesis rate; and enhancement of protease activity [2–4]. Plants have developed various strategies to cope with Cd toxicity, such as efflux chelation, sequestration, or detoxification [9]. The complex molecular network in response to Cd stress comprises principally: osmoregulation, stimulation of antioxidant defense system, over-production of signaling molecules, ion homeostasis, and stimulation of transporters [2, 7, 10, 11]. Transcriptome analyses have revealed that transcription factors (e.g., MYBs, WRKY, EP2/ERF, etc.) are essential for tolerance to Cd exposure [7, 8, 12, 13]. Furthermore, these studies have shown that the Cd stress response of plants varies with species, varieties, growth conditions, and duration.

*Hibiscus syriacus* L., a member of the Malvaceae family, is an important ornamental shrub. It is mainly distributed in South and East Asia, where its flowers, fruits, roots, skin, and stem are extensively used in traditional medicine to cure several diseases [14–16]. For instance, studies have shown that thethe plant possesses diverse pharmacological properties such as anti-melanogenesis [17], anti-osteoporosis [18], anti-ultraviolet B-induced damages [19], anti-depressant and neuroprotective [14, 16], antioxidation [15], and anti-inflammation [20]. Globally, *Hibiscus spp.* are highly tolerant to abiotic stress. However, compared with other *Hibiscus spp.*, few studies have focused on the response of *H. syriacus* to abiotic stress, especially heavy metals’ accumulation, and tolerance mechanisms [12, 21]. Yang et al. have evaluated the physiological response of three *H. syriacus* varieties (*f. paeoniflorus*, Hongxing, and *f. albus-plenus*) under different Cd concentrations and identified the variety “Hongxing” as a potential Cd tolerant [22]. However, only one antioxidant enzyme activity was recorded. Hence, it is essential to gain insight into the molecular mechanisms involved in Cd tolerance of *H. syriacus* to provide resources for its possible use in phytoremediation or minimizing human Cd ingestion from its organs.

In this study, we re-evaluated the physiological response of “Hongxing” under high Cd treatment. Through transcriptome analysis, we identified the differentially expressed genes and filtered out some key genes (including transcription factor family genes) that can be targeted to improve the Cd tolerance of *H. syriacus*. In addition, we revealed the major pathways underlying Cd tolerance in Hongxing. Our results deepen our understanding of Cd stress tolerance in *H. syriacus* and provide important resources for future research.

## Results

### Effect of high Cd concentration on plant growth and chlorophyll content

**Fig. 1:**
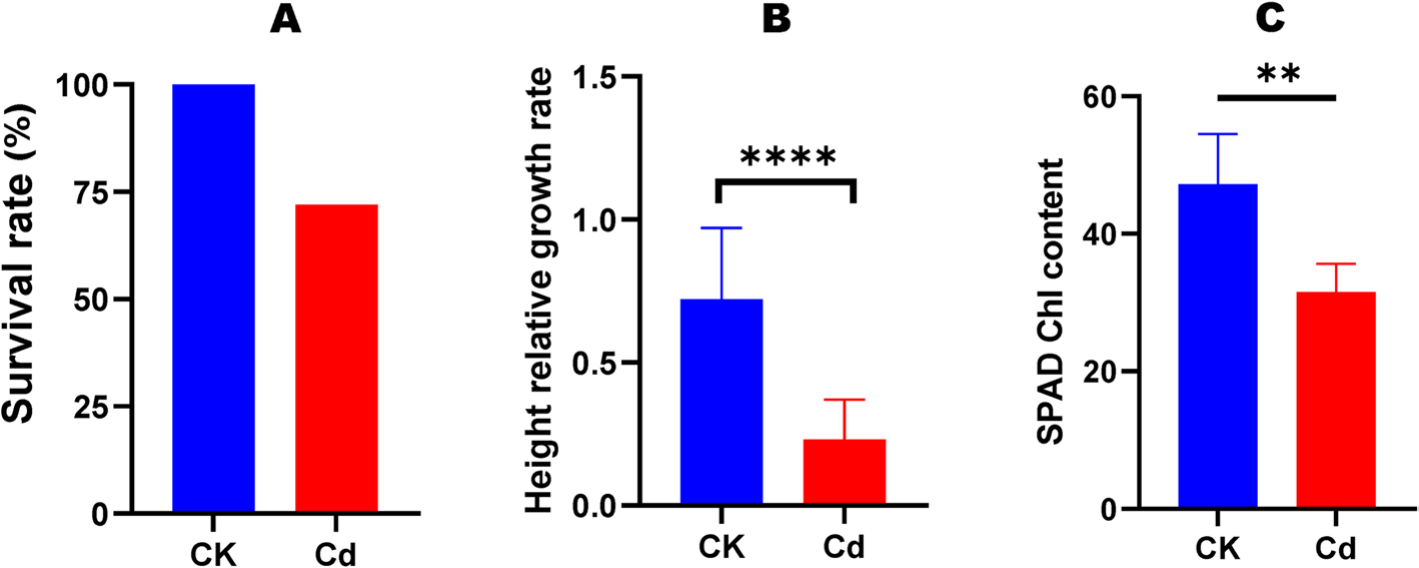
Effect of high concentration of Cd on *H. syriacus* growth and chlorophyll content. **A** Survival rate; **B** Height relative growth rate; **C** Chlorophyll content. CK, control; Cd, stressed. Values are means ± SD (*n* = 3). ** *P* < 0.01; **** *P* < 0.0001, t-test

To determine the impact of the Cd treatment on the development of *H. syriacus* plants, we analyzed the survival rate, height relative growth rate (HRGR), and chlorophyll content (Fig. 1). Compared to the control plants (CK), the survival rate of Cd-treated plants was reduced by 28%, supporting that this variety can tolerate high Cd stress (Fig. 1A). The HRGR and chlorophyll content of Cd-treated plants were significantly reduced compared to CK (Fig. 1B, C), indicating that Cd treatment affected the global metabolism.

### Lipid peroxidation and antioxidant enzymes status

**Fig. 2:**
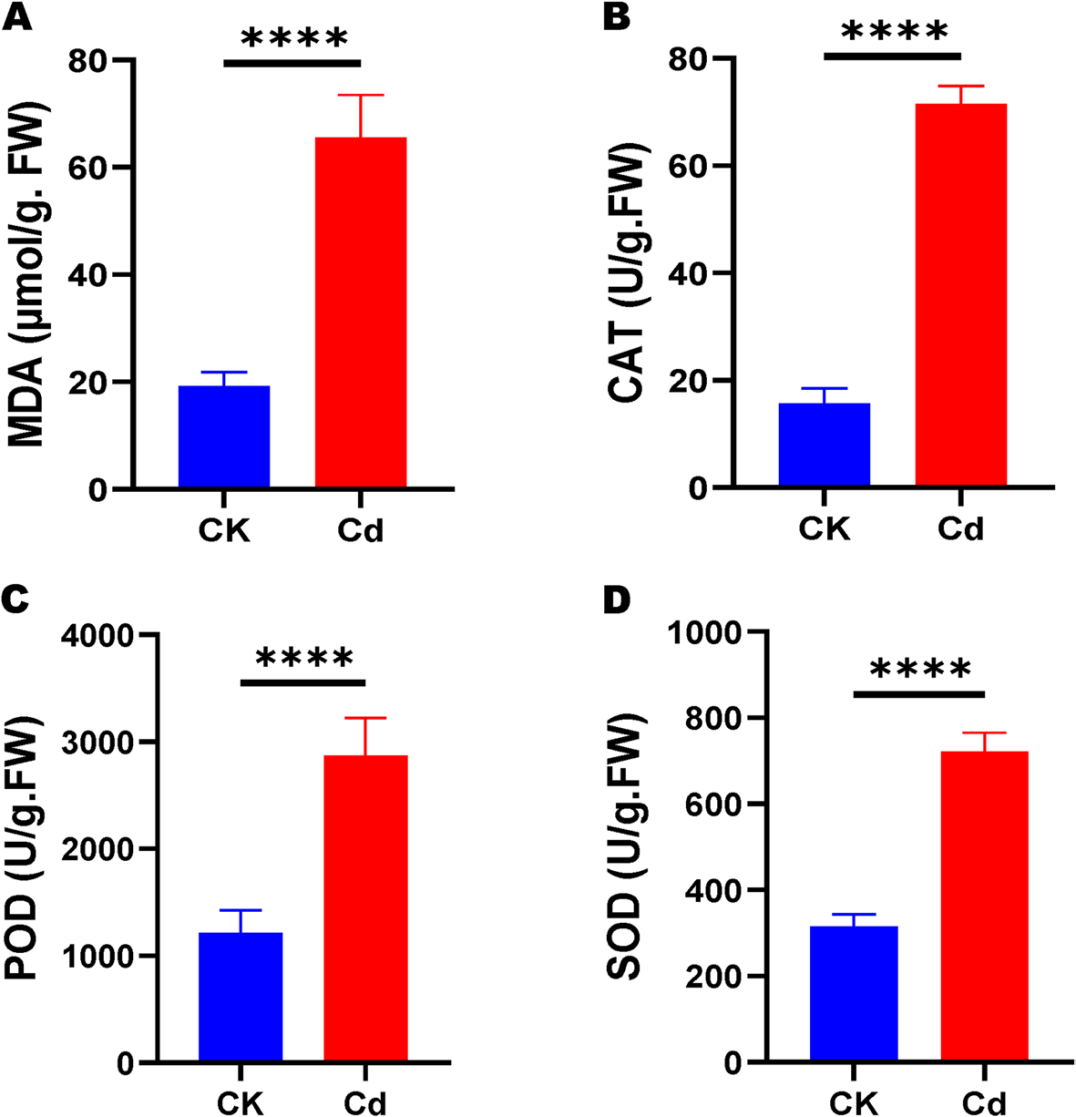
Effects of high concentration of Cd on MDA content (**A**), and activities of CAT (**B**), POD (**C**), and SOD (**D**) in *H. syriacus* leaves. Values are means ± SD (*n* = 3). Bars indicate SD. **** *P* < 0.0001, t-test

The malondialdehyde (MDA) content reflects the degree of lipid peroxidation and the status of biological membranes when subjected to oxidation stress by ROS. The MDA content of Cd-treated plants was significantly increased compared to CK, indicating that Cd stress has damaged membranes’ structure and functions. For instance, the MDA content of Cd-treated plants was 3.38-fold higher than in CK (Fig. 2A).

We further investigated the activities of antioxidant enzymes, including CAT, POD, and SOD, to confirm the Cd-induced oxidative stress in *H. syriacus* plants. The results showed the activities of antioxidant enzymes were significantly induced by the Cd stress (Fig. 2B, C, D). Compared to the control, CAT, POD, and SOD activities were 4.57, 2.36, and 2.29-fold higher in Cd-treated plants, respectively.

### Transcriptome analysis of CK and Cd-treated plants

In order to reveal the molecular mechanisms involved in Cd stress response in *H. syriacus*, leaf samples from control (CK-d0) and Cd-treated plants (Cd-d0) were collected immediately after the stress application for total RNA extraction and sequencing. In addition, the Cd-treated plants were sampled ten days after the end of Cd stress induction (Cd-d10) end to investigate the molecular changes following exposure to Cd stress in *H. syriacus*. The sequencing yielded 42,769,970 to 49,156,662 bp and 41,587,697 to 47,717,936 bp raw and clean reads, respectively (Table 1). We observed strong correlations (*r* > 0.94) between samples of the same group (Supplementary Fig. 1). The error rate of all samples was 0.03%, and the Q20 and Q30 values were greater than 97 and 92%, respectively. The GC content for all samples ranged from 44.28 to 44.66 (Table 1). These results indicated that the sequencing data were of high quality.

**Table 1:** Summary of throughput and quality Illumina-based transcriptome sequencing of *H. syriacus* leaves under high concentration Cd treatment

Sample	Raw Reads (bp)	Clean Reads (bp)	Clean Base (G)	Error Rate (%)	Q20 (%)	Q30 (%)	GC Content (%)
CK-d0-1	46,842,252	44,941,528	6.74	0.03	97.74	93.57	44.53
CK-d0-2	43,070,706	41,769,672	6.27	0.03	97.7	93.47	44.28
CK-d0-3	46,752,126	45,214,420	6.78	0.03	97.68	93.46	44.66
Cd-d0-1	45,030,500	43,619,138	6.54	0.03	97.71	93.48	44.52
Cd-d0-2	44,987,600	43,637,464	6.55	0.03	97.28	92.44	44.44
Cd-d0-3	44,315,790	42,691,762	6.4	0.03	97.22	92.2	44.39
Cd-d10-1	43,148,652	41,925,432	6.29	0.03	97.13	92.02	44.66
Cd-d10-2	42,769,970	41,587,692	6.24	0.03	97.25	92.41	44.4
Cd-d10-3	49,156,662	47,717,936	7.16	0.03	97.08	92.05	44.62

We then de novo assembled the high-quality clean reads into unigenes using the Trinity software [23, 24]. The transcripts and unigenes exhibited similar lengths (Supplementary Fig. 2), with an average length of 835 and 853, respectively, and an N50 value of 1,227 and 1,239, respectively. Sequence similarity analysis showed that 71.21% (216,347) of unigenes were at least highly identical to known proteins in seven different databases. Particularly, 47.47, 70.45, 48.04, 70.21, 39.33, 59.83, and 37.25% of the unigenes were annotated in the KEGG, NR, Swiss-Prot, Trembl, KOG, GO, and Pfam databases, respectively. Regarding other species, 60.69% of the unigenes showed high similarity with sequences in *Grossypium spp.*, the same family members (Supplementary Fig. 3).

For an overview of the degree of similarity of the transcriptome of the different samples, we conducted the principal component analysis (PCA) and hierarchical clustering analysis (HCA). On the HCA and PCA plots, samples in the same group clustered together, confirming the reproducibility of the experiment (Supplementary Fig. 4A, B). CK-d0, Cd-d0, and Cd-d10 samples were completely separated, indicating that their transcriptomes were very different and differentially expressed genes (DEGs) could be identified.

### Cd-induced DEGs in *H. syriacus*

**Fig. 3:**
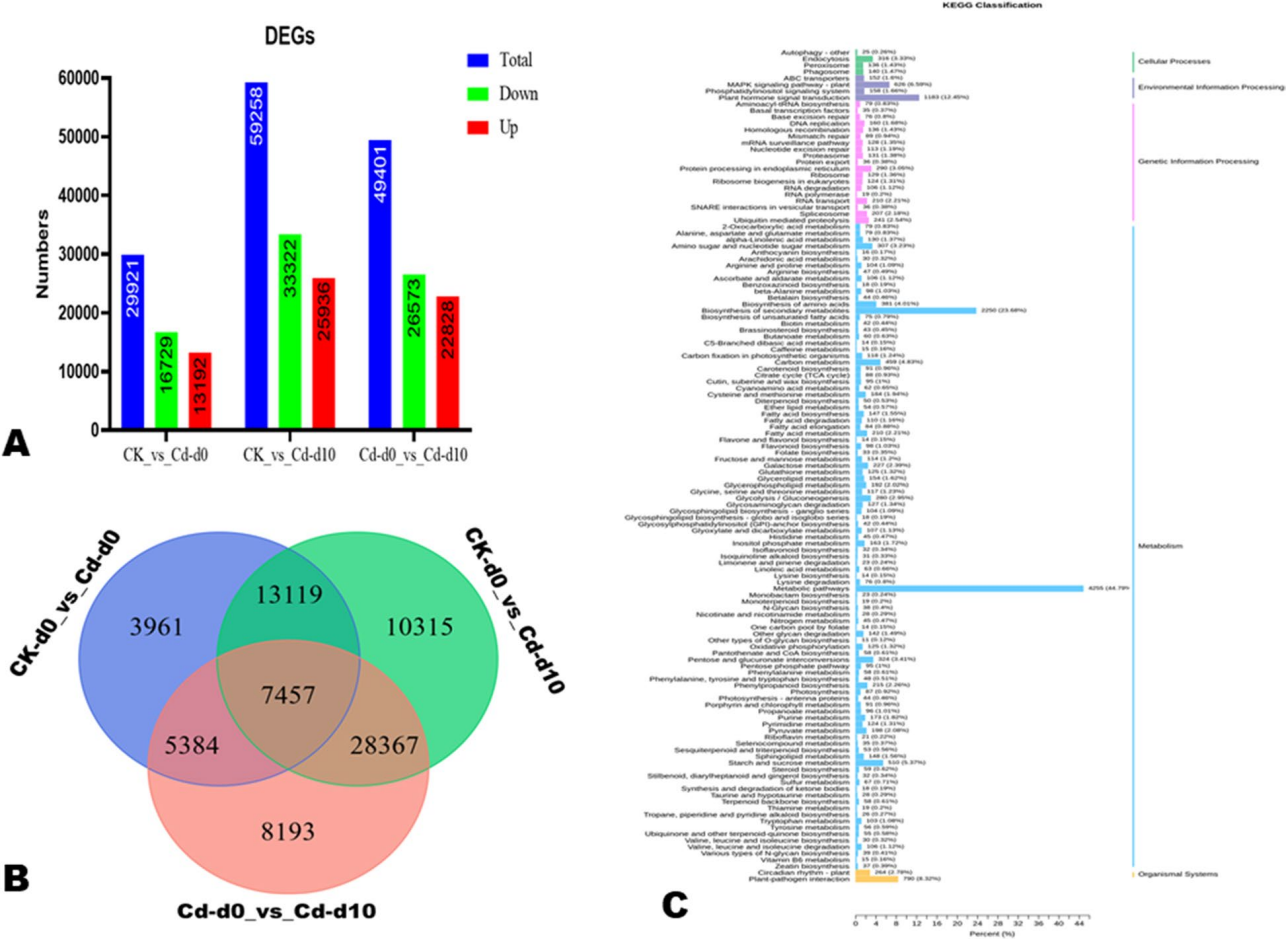
DEGs in *H. syriacus* leaves under Cd stress and functional analysis. **A** Summary of the number of DEGs between the different groups; **B** Venn diagram showing the common DEGs between control (CK-d0), stressed plants at the end of Cd treatment (Cd-d0) and ten days after the end (Cd-d10); **C** KEGG annotation results of the DEGs between CK-d0 and Cd-d0

By applying the criteria of threshold |log2Fold Change|≥ 1 and *p*-value < 0.5, we filtered out the DEGs between the different groups. The volcano plots are shown in Supplementary Fig. 5. There were 29,921 DEGs, including 16,729 down-regulated and 13,192 up-regulated between CK-d0 and Cd-d0 (Fig. 3A), indicating the Cd stress influenced significantly *H. syriacus* plants’ metabolisms. Meanwhile, between Cd-d0 and Cd-d10, we identified 26,573 and 22,828 down- and up-regulated genes, respectively (Fig. 3A), suggesting different mechanisms were initiated by the plant to recover from the Cd stress. Between CK-d0 and Cd-d10, there were 59.258 DEGs, including 33.322 and 25.936 down- and up-regulated, respectively (Fig. 3A). Venn diagram showed that there 7,457 genes were differentially expressed in CK-d0, Cd-d0, and Cd-d10 (Fig. 3B).

### Molecular mechanisms involved in Cd stress response in *H. syriacus*

To provide an overview of the Cd stress-responsive molecular mechanisms in *H. syriacus*, we performed GO and KEGG annotation and enrichment analysis of DEGs between CK-d0 and Cd-d0 (Table S2). The Functional annotation and enrichment analyses revealed that the main molecular mechanisms involved in Cd stress tolerance in *H. syriacus* were transport, plant hormone signal transduction, MAPK signaling pathway, antioxidant process, maintenance of membranes’ structure and properties, sulfur and nitrogen metabolism, and Cd homeostasis and detoxification (Fig. 3C; Supplementary Fig. 6).

### Heavy metal transport associated DEGs under Cd stress

**Fig. 4:**
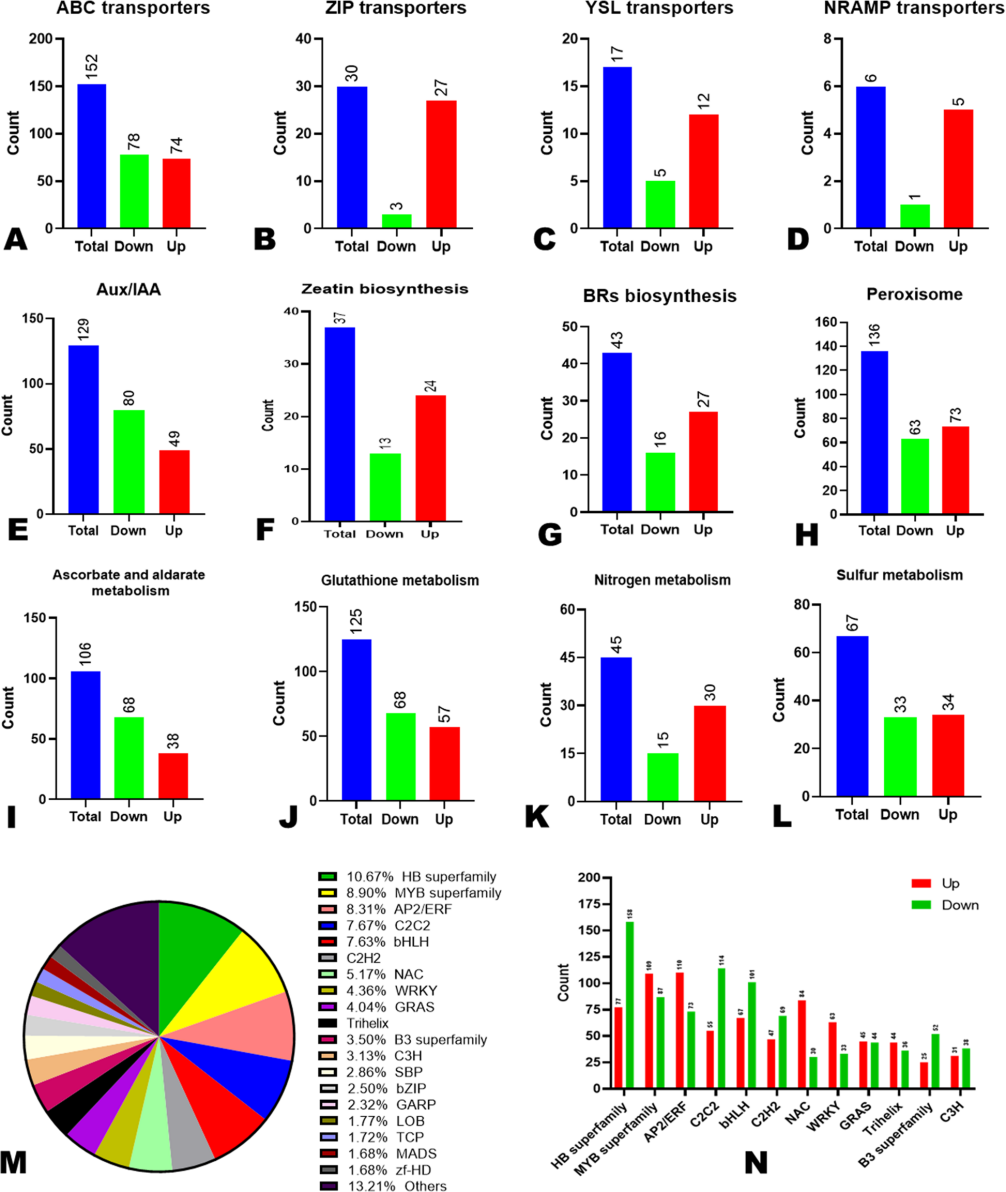
Overview of DEGs associated with some key Cd tolerance-related pathways and differentially expressed transcription factors (DETFs). **A**-**D** Heavy metal transporters; **E**–**G** Some phytohormones; **H**-**J** Antioxidant system; **K** Nitrogen metabolism; **L** Sulfur metabolism; **M** Distribution DETFs between CK-d0 and Cd-d0.; **N** Number of up- and down-regulated DETFs of major identified TFs

Heavy metal transporters, such as ABC (ATP-binding cassette transporter), yellow stripe 1-like (YSL), zinc transport protein (ZIPs), and NRAMP (natural resistance-associated macrophage protein), play critical roles in Cd uptake and transport. A total of 152 (74 up-regulated), 30 (27 up-regulated), 17 (12 up-regulated), and 6 (5 up-regulated) differentially expressed ABC, ZIP, YLS, and NRAMP DEGs, respectively, were identified in “Hongxing” plant leaves (Fig. 4A-D). There were more up-regulated ZIP, YLS, and NRAMP transporter family genes, suggesting they might play essential roles in *H. syriacus* Cd tolerance mechanisms. Although there were more down-regulated ABC family genes, six of the up-regulated ones, including *Cluster-15126.17907* (ABCG22), *Cluster-15126.156181* (ABCG36), *Cluster-15126.153662* and *Cluster-15126.128716* (ABCG11), *Cluster-15126.170014* (ABCB27), and *Cluster-15126.273326* (ABCC9) were highly induced (|log2Fold Change|≥ 6) under the Cd stress (Table S2). These genes might also play essential roles in *H. syriacus* Cd tolerance mechanisms.

### Antioxidant system-associated DEGs

A robust antioxidant system is required for plant Cd tolerance. We screened the DEGs and found that 136 (73 up-regulated), 106 (38 up-regulated), and 125 (57 up-regulated) were related to peroxisome, ascorbate and aldarate metabolism, and glutathione metabolism, respectively (Fig. 4H-J). One DEG encoding SOD (*Cluster-15126.83939*), one encoding ascorbate peroxidase (*Cluster-15126.82748*), two encoding glutathione reductase (*Cluster-15126.171365* and *Cluster-15126.48873*), sixteen encoding glutathione-S-transferase, and one encoding phospholipid hydroperoxide glutathione peroxidase (*Cluster-15126.156132*), were up-regulated under Cd stress. It is known that some proteins, such as heat shock proteins (HSPs) and late embryogenesis abundant (LEA) proteins, positively regulate the antioxidant system to enhance Cd tolerance [25–27]. We then examined the expression patterns of differentially expressed HSP and LEA genes (Supplementary Fig. 7; Table S2). Of the 18 and 60 differentially expressed HSP and LEA genes, 14 and 43 were up-regulated, respectively, implying these proteins might play important roles in Cd tolerance in *H. syriacus.*

### Sulfur and nitrogen metabolisms associated DEGs

Nitrogen and sulfur metabolism could improve plants’ Cd tolerance via the production of chelating agents such as nitric oxide, phytochelatins (PCs), glutathione (GSH), and metallothioneins (MTs) [13, 28]. 66.66% (30 out of 45) and 50.74% (34 out of 67) of the DEGs associated with nitrogen and sulfur metabolism, respectively, were up-regulated (Fig. 4K, 4L). Some key genes in these pathways, including two nitric oxide biosynthetic genes (*Cluster-15126.265962* and *Cluster-15126.123193*), eight glutamine synthetase (*Cluster-15126.145221*, *Cluster-15126.155917*, *Cluster-15126.155919*, *Cluster-15126.161102*, *Cluster-15126.163874*, *Cluster-15126.168087*, *Cluster-15126.186909*, and *Cluster-15126.241616*), three serine acetyltransferase 1 (*Cluster-15126.159268*, *Cluster-15126.229215*, and *Cluster-15126.229216*), one serine acetyltransferase 3 (*Cluster-15126.259051*), one serine acetyltransferase 5 (*Cluster-15126.87922*), three ATP sulfurylase 1 (*Cluster-15126.136827*, *Cluster-15126.136830*, and *Cluster-15126.44008*), two ATP sulfurylase 2 (*Cluster-15126.102668* and *Cluster-15126.102669*), and one persulfide dioxygenase (*Cluster-15126.118587*), were up-regulated (Table S2).

### DEGs associated with plant hormone signal transduction and MAPK signaling pathway

A total of 1,183 and 626 DEGs were assigned to the plant hormone signal transduction and MAPK signaling pathway, respectively (Fig. 3C; Supplementary Fig. 8), indicating the importance of these pathways in modulating the Cd tolerance molecular mechanisms in *H. syriacus*. The auxin (IAA) biosynthesis pathway-related genes were the most identified with 129 DEGs, of which 49 were up-regulated (Fig. 4E). Other main plant hormones, including cytokinin, ethylene, gibberellin, abscisic acid (ABA), and brassinosteroids (BRs) biosynthetic genes were significantly activated in the leaves (Table S2). Compared to the down-regulated genes, there were more up-regulated genes in zeatin (cytokinin) and BRs biosynthesis pathways, respectively (Fig. 4F, G), suggesting these phytohormones are essential for Cd tolerance of “Hongxing” plants.

As TFs play important roles in coordinating signaling pathways and plant abiotic and biotic stress responses, we filtered out the differentially expressed TFs (DETFs) between CK-d0 and Cd-d0 to allow an overview of major TFs that modulate Cd stress response in *H. syriacus*. In total, 2,203 DETFs were identified (Table S3). The major DETFs included HB superfamily (10.67%), MYB superfamily (8.90%), EP2/ERF (8.31%), C2C2 (6.67%), bHLH (7.63%), C2H2 (5.27%), NAC (5.17%), WRKY (4.36%), and GRAS (4.03%) (Fig. 4M). Compared to down-regulated DEGs, the up-regulated MYB, AP2/ERF, NAC, and WRKY were more (Fig. 4N), indicating they might play critical roles in Cd tolerance in *H. syriacus*.

### DEGs associated with Cd homeostasis and detoxification

**Fig. 5:**
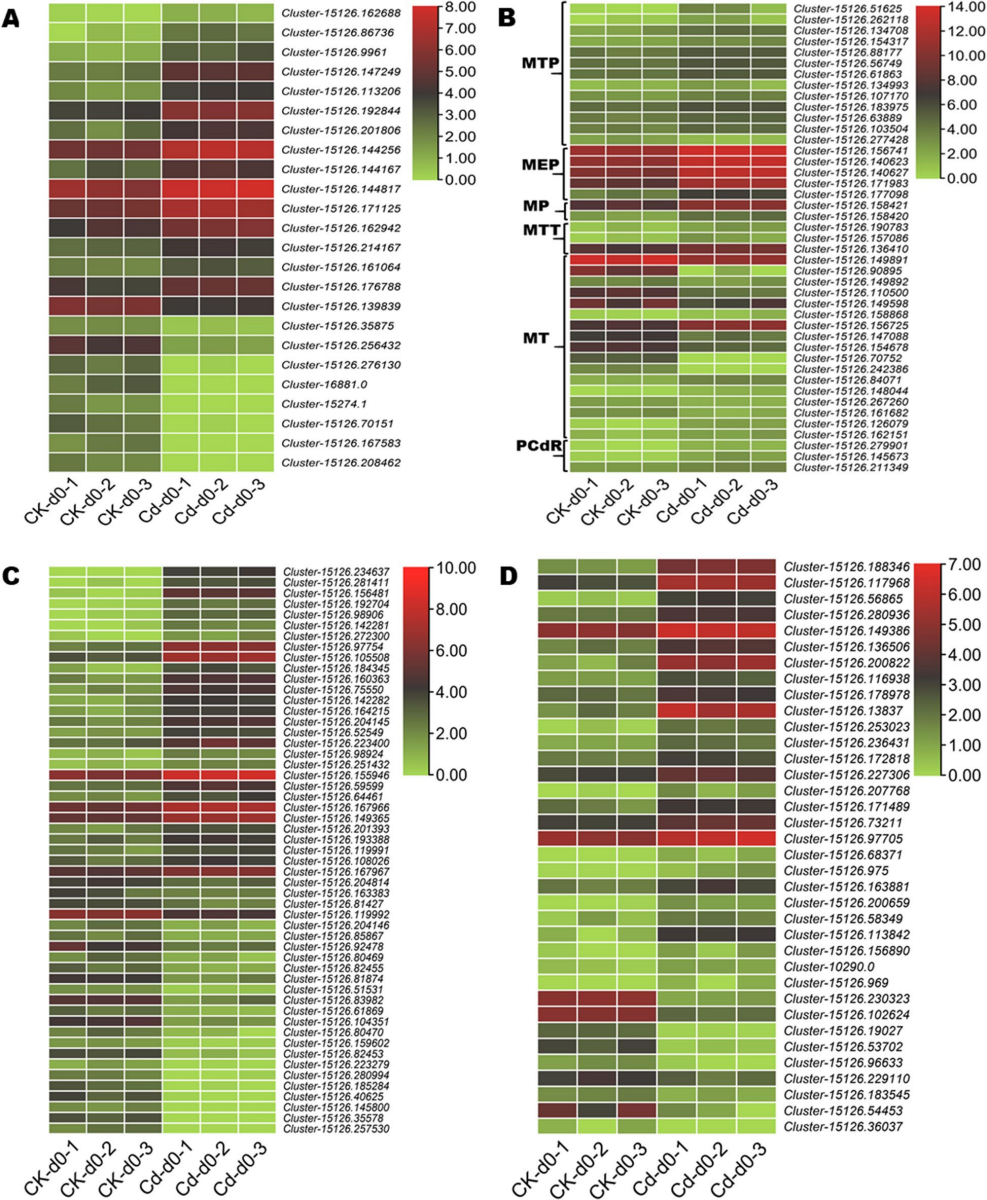
Heatmap of log2FC values of the DEGs enriched in heavy metal-associated plant proteins, HPPs (**A**), heavy metal-associated isoprenylated plant proteins, HIPPs (**C**), other metal homeostasis and detoxification-related genes (**B**), and detoxification protein (**D**). MTP, metal tolerance protein; MT, metallothionein; MEP, metalloendoproteinase; MTT, metallothiol transferase; MP, metallophosphoesterase and metallo-independent phosphoresine phosphatase; PCdR, plant Cd resistance protein

Cd homeostasis and detoxification are key Cd tolerance mechanisms in plants. These mechanisms are mainly driven by heavy metal-associated plant proteins (HPPs), heavy metal-associated isoprenylated plant proteins (HIPPs), metal tolerance proteins (MTPs), metallothionein family genes (MTs), metalloendoproteinase family genes (MEPs), metallothiol transferase genes (MTTs), metallophosphoesterase and metallo-independent phosphoresine phosphatase (MPs), and plant Cd resistance proteins (PCdRs). In total, 120 main Cd homeostasis and detoxification-related genes were differentially expressed in “Hongxing” plant leaves (Table S4). Their expression patterns are shown in Fig. 5A-C. They included twenty-seven (27 out of 53), fifteen (15 out of 24), twelve (12 out of 13), twelve (12 out of 17), and three (3 out of 3) up-regulated HIPPs, HPPs, MTPs, MTs, and PCdRs genes, respectively. It was noteworthy that three HIPPs (*Cluster-15126.234637*, *Cluster-15126.156481,* and *Cluster-15126.281411*) and one MTP (*Cluster-15126.51625*) were significantly induced (|log2Fold Change|≥ 6) under the Cd stress. Two PCdRs (*Cluster-15126.279901* and *Cluster-15126.145673*) were induced by 4.54 and 4.04-folds, respectively.

Besides, we have identified 36 DEGs, including 27 up-regulated and nine down-regulated genes that encode protein detoxification (Fig. 5D). These genes might also contribute to Cd detoxification to enhance Cd tolerance of “Hongxing” plants.

### Changes in molecular mechanisms after exposition to Cd stress in *H. syriacus*

To understand how *H. syriacus* plants recover from Cd stress, we carried out GO and KEGG annotation and enrichment analyses on the DEGs between Cd-d0 and Cd-d10 (Table S5). The functional analyses indicated that the DEGs were mainly related to antioxidant and developmental processes (Supplementary Figs. 9 and 10), indicating the plants have maintained the tolerance mechanisms and initiated the biosynthesis of novel compounds to sustain growth and development. For instance, most GO terms related to the biological process were amino acids metabolism and transport, cellular biogenic amine metabolic process, transmembrane drug transport, hydrogen peroxide catabolic process, response to high light intensity, lateral root formation, and xyloglucan metabolic process (Supplementary Fig. 9). In the cellular component, the primary GO term was cell surface. Oxidoreductase activity, peroxidase activity, solute: cation symporter activity, and sugar transmembrane transporter activity were the major GO terms in the molecular function (Supplementary Fig. 9). The KEGG annotation and enrichment analysis assigned the DEGs between Cd-d0 and Cd-d10 mainly to the biosynthesis of secondary metabolites, plant hormone signal transduction, carbon metabolism, pyruvate metabolism, and amino acids metabolism (Supplementary Fig. 10). Noteworthy, 168 and 66 DEGs were assigned to lateral root formation and water channel activity, respectively (Supplementary Fig. 9), inferring that plants have improved their root system to enhance nutrients and water uptake.

### qRT-PCR validation and schematic representation of the Cd stress tolerance mechanisms

**Fig. 6:**
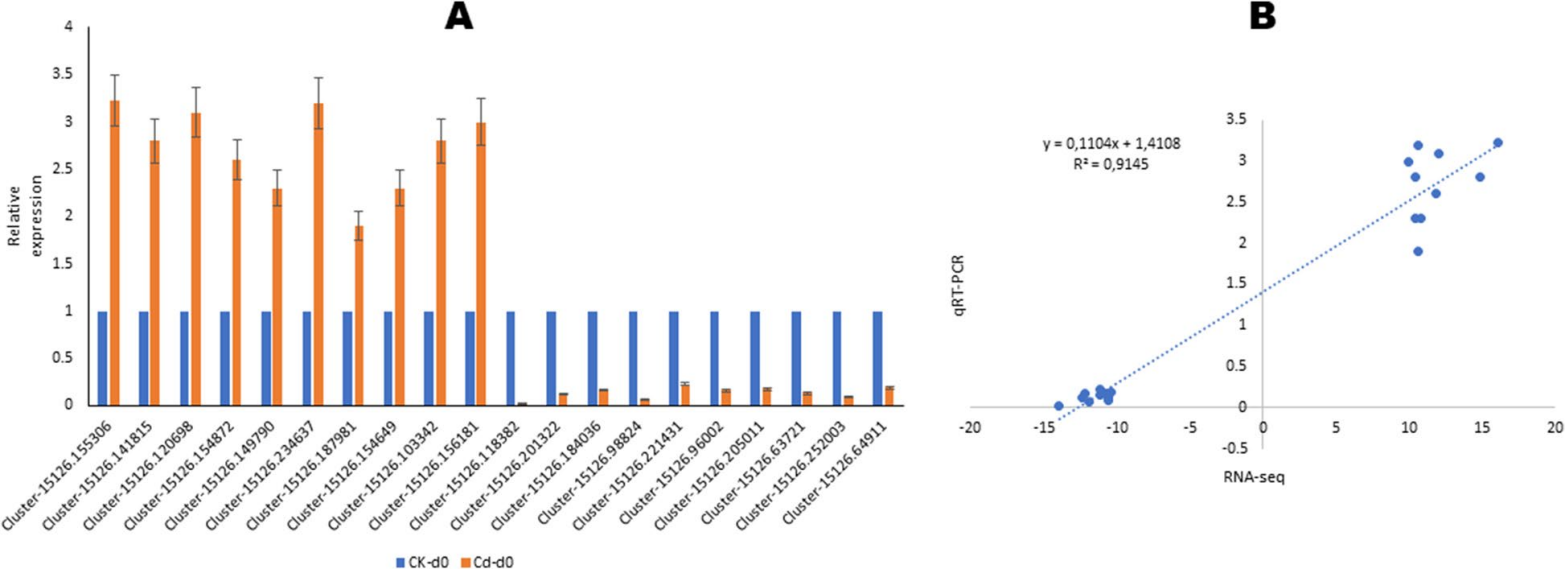
qRT-PCR validation of twenty (20) selected most differentially regulated genes

**Fig. 7:**
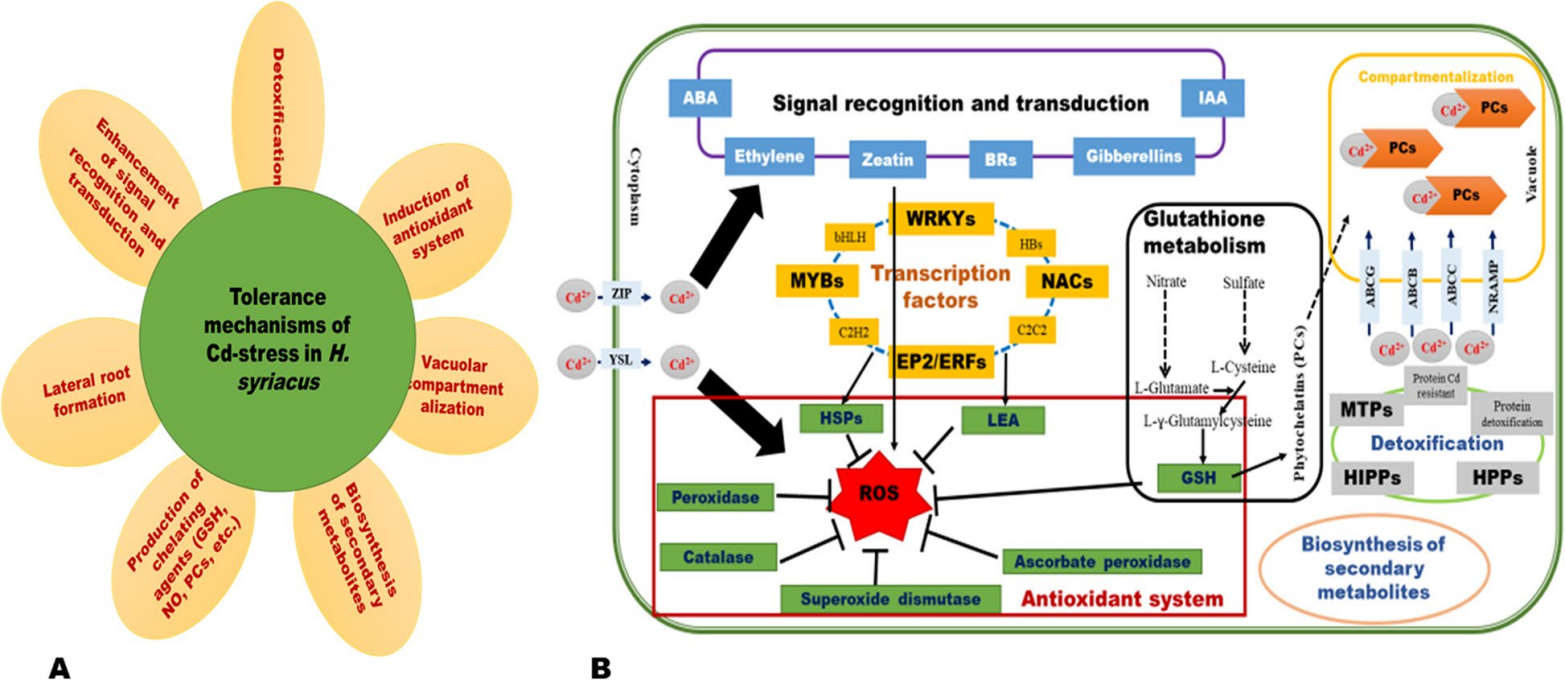
Graphical representation of the Cd stress tolerance mechanisms in *H. syriacus* leaf. **A** Major tolerance mechanisms. **B** Schematic diagram of gene expression network

To verify the reliability of the RNA-seq data, we randomly selected 20 most deregulated DEGs (|log2Fold Change|≥ 10), including ten up-regulated and ten down-regulated between CK-d0 and Cd-d0, for qRT-PCR analysis. As a result, the expression patterns of these genes by qRT-PCR were consistent with the transcriptome analysis (*r*^2^ = 0.9145, Fig. 6), confirming that the RNA-seq data and our findings are reliable. Therefore, we constructed a graphic representation of the Cd stress tolerance mechanisms in *H. syriacus* leaves to enable a global overview of our results (Fig. 7).

## Discussion

Understanding the molecular mechanisms involved in the regulatory network of Cd tolerance in plants is a prerequisite for identifying potential plant species for phytoremediation and minimizing the risk of chronic disease in humans. RNA-seq analysis has been widely applied to reveal the molecular basis of Cd tolerance in many crops [8, 12, 29, 30]. This study analyzed the tolerance mechanisms of *H. syriacus* under high Cd concentration for the first time.

Cd stress is one of the most damaging abiotic stresses affecting chloroplast ultrastructure and nutrient uptake from soil [3, 4, 31]. This results in reduced chlorophyll synthesis and photosynthetic efficiency, ultimately leading to drastic growth retardation or plant death [3, 4, 31]. In agreement with these reports, the high Cd treatment significantly inhibited the height growth and chlorophyll synthesis of *H. syriacus* plants. However, only 28% of the plant died, supporting that “Hongxing” is tolerant to high Cd stress. Moreover, these results infer that “Hongxing” can perform well when cultivated on soils with low to moderate Cd concentrations. Similar results have been observed for kenaf (*H. cannabinus* L.), a potential heavy metal phytoremediation plant [12]. These findings suggest that *H. syriacus* could also be used for phytoremediation in Cd-contaminated soils. However, it is necessary to evaluate Cd accumulation in different organs of the plant under various Cd concentrations to reduce the risk of human contamination, as this species is intensively used in traditional medicine.

Previous studies have shown that Cd stress enhances the production of ROS, which alters the structure of biological membranes’ structure, damages cellular organelles, alters biomolecules (DNA, RNA, lipids, proteins, etc.), and ultimately disrupts the normal metabolism and development of plants [11, 12, 31–33]. We found that the high-level Cd treatment significantly enhanced the accumulation of MDA, which is a product of lipid peroxidation by ROS. Plants scavenge ROS by stimulating antioxidant enzyme activities, inducing the biosynthesis of antioxidant compounds such as glutathione and ascorbate in chloroplasts, and up-regulating antioxidant system-related genes [13, 32, 34]. Herein, we also observed a significant increase in the activities of CAT, POD, and SOD by 4.57, 2.36, and 2.29-fold, respectively, compared to the control. Supportively, numerous antioxidant-related genes, including peroxidases, cytochrome P450, and glutathione-S-transferase, were induced more than sixfold in Cd-d0 compared to CK-d0. These findings imply that CAT and POD may be essential for Cd tolerance in *H. syriacus*, and that targeting CAT and POD-related genes may be efficient in improving the Cd tolerance capacity of the plant. Similar results have been reported in Kenaf [12].

Plant growth regulators, including phytohormones, polyamines, nitric oxide, and brassinosteroids, and numerous transporters, such as ABC, NRAMP (natural resistance-associated macrophage protein), ZIPs (ZRT, IRT-like proteins), CDFs (cation diffusion facilitators), MATE efflux family proteins, and CAX (cation exchangers), have been reported to play important roles in plant tolerance to heavy metals [28, 35, 36]. It has been reported that Cd transport and perception can activate signaling cascades in plants, and the Cd-induced signaling is associated with both exogenous and endogenous levels of plant growth regulators [35]. In this study, the DEGs were mainly enriched in transport, plant hormone signal transduction, and MAPK signaling pathway. We identified several plant hormone pathways-related DEGs, of which auxin-related DEGs were the most abundant. There were more up-regulated cytokinin, BRs, and Ethylene biosynthesis genes. These results indicate that phytohormones are essential for Cd stress tolerance in *H. syriacus,* and their exogenous application could be an alternative to improve the heavy metal tolerance capacity of the plant. The influence of heavy metal-induced stress on MAPK signaling has been demonstrated in previous studies [12, 31, 37]. These reports indicated that the MAPK signaling pathway is activated by heavy metals for the coordinated regulation of diverse cellular metabolic processes, including the modulation of hormone signaling and the regulation of some specific TFs. Many up-regulated heavy metal transporter DEGs were identified, including ABC, YLS, NRAMP, and ZIP family genes. Particularly, we identified six highly induced (|log2Fold Change|≥ 6) ABC transporter family genes, including *Cluster-15126.17907* (ABCG22), *Cluster-15126.156181* (ABCG36), *Cluster-15126.153662* and *Cluster-15126.128716* (ABCG11), *Cluster-15126.170014* (ABCB27), and *Cluster-15126.273326* (ABCC9). The ABC transporter family is the largest transporter group. It plays essential roles in diverse cellular metabolic processes such as osmotic homeostasis, nutrient uptake, fatty acid import, hormone transport, and also Cd tolerance [38, 39]. Five differentially expressed ABC transporter family genes have been identified in kenaf [12]. Most of the YLS, NRAMP, and ZIP DEGs were up-regulated, indicating they might be essential for Cd uptake and transport in *H. syriacus*. Further studies are required to elucidate the specific roles of these biomolecules and the MAPK pathway in Cd transport, sequestration, and detoxification.

Transcription factors (TF) play critical roles in regulating abiotic and biotic stresses. In rice, several Cd stress-induced WRKY, NAC, MYB, and AP2 TF family genes were identified through DEGs analysis [5, 10]. In this study, the number of up-regulated MYB, NAC, AP2/ERF, and WRKY family genes was higher than the number of down-regulated genes, indicating that these TFs might play key roles in the regulatory network of Cd stress tolerance in *H. syriacus*. Other key TFs were also identified, including C2H2, GRAS, HB superfamily, bHLH, etc., supporting the importance of TFs for a coordinated tolerance response to Cd stress in *H. syriacus*. Interestingly, the gene *Cluster-15126.149216*, which is a C2H2-like zinc finger TF family gene, was induced 6.5-fold in Cd-d0 compared to CK-d0. Its *Arabidopsis* homolog *AtZAT6* in enhances Cd tolerance via the glutathione-dependent pathway [40]. Furthermore, we screened out the three most up-regulated heavy metal-associated isoprenylated plant protein (HIPP) genes (*Cluster-15126.234637, Cluster-15126.156481, and Cluster-15126.281411)* and one metal tolerant protein (MTP) (*Cluster-15126.51625*) that may considerably contribute to Cd tolerance of *H. syriacus*. HIPPs contain a metal binding domain (HMA) and a C–terminal isoprenylation motif (metallochaperones) and are found in vascular plants only [41]. They play critical roles in plant responses to various stresses, specifically in heavymetal homeostasis and detoxification [41]. The HPPs gene family has been extensively characterized in rice and *Triticeae* species [42, 43]. Functional characterization of these genes is required to decipher the regulatory network of Cd stress response and to exploit their potential for improving heavy metal tolerance in *H. syriacus* and other crops. The validation of the identified potential candidate genes will clarify their functional roles, regulatory pathways, and protein expression levels. In addition, it will provide markers for targeted improvement of crop Cd tolerance. Functional genomics integrates cell biology and molecular biology studies to understand the overall structure, regulation, and function of a gene [44].

Besides, the analysis of the DEGs between Cd-d0 and Cd-d10 showed that the tolerance mechanisms were maintained ten days after the end of the Cd treatment. In addition, we noticed a stimulation of anabolism, the formation of novel lateral roots, and the activation of water channels. These results indicate that the plant has initiated additional pathways to stimulate Cd detoxification and enhance growth and development. Furthermore, the formation of novel roots may enhance nutrient uptake and help to correct Cd^2+^-induced morpho-physiological damages.

## Conclusions

In summary, this study unveiled the molecular mechanisms involved in Cd stress tolerance of *H. syriacus*. Very low mortality of “Hongxing” plants was observed after high-concentration Cd treatment (30 days). This was achieved by enhancing their antioxidant system, plant hormone signal transduction, MAPK signaling pathway, molecules transport, sulfur and nitrogen metabolism, Cd homeostasis and detoxification, secondary metabolism, DNA and nucleosome processes, and fatty acid metabolism. Through transcriptome sequencing and analysis, DEGs were identified, of which some key genes were screened out for targeted improvement of Cd stress tolerance of *H. syriacus* based on previous studies in other crops. Some MYB, AP2/ERF, NAC, WRKY, NRAMP, YSL, ZIP, and ABC family genes may play critical roles in Cd stress tolerance of *H. syriacus*. Our results deepen the understanding of Cd stress response in *H. syriacus* and offer important resources for future research to improve the heavy metal tolerance capacity of crops.

## Materials and methods

### Plant material and growth condition

The Cd-tolerant *Hibiscus syriacus* variety “Hongxing” identified previously was used in this study [22]. The plant material is available at the Key Laboratory for Forest Resources Conservation and Utilization in the Southwest Mountains of China under the accession number: ZHY0045A22. The formal identification was conducted by Prof Lanlan Liu. No permission is required to collect and study the plant material. The experiment was conducted in a completely randomized manner in pots of 25 cm × 13 cm × 16 cm dimensions. The pots were filled with 2 kg of sterilized (at 121 °C for 60 min) soil taken from Hunan Forest Botanical Garden. Two-year-old plants were adequately isolated from the same forest and transplanted in the pots, one in each. The experiment was conducted from June to September 2021 on the roof of the Zhongnan Forestry Department, College of Landscape Architecture, University of Technology. The plants were watered normally, and after 30 days, the ones in good conditions with consistent growth were selected for the Cd stress treatment. An 8 g/L CdCl_2_.2.5H_2_O solution was prepared and applied every two days to the pots until the Cd concentration of the soil reached 400 g/kg. One batch of the plants (control plants) received only water without Cd (CK). The treatment lasted 30 days. The experiment was conducted with three replicates, each containing fifteen (15) plants. After the Cd treatment, all the plants were watered (without Cd) regularly.

### Evaluation of physiological traits

The survival rate (SR) was evaluated using the following formulae: SR = (number of survived plants/numbers of initial plants) × 100. At the end of the Cd treatment (d0) and 60 days after the end of the treatment (d60), the survived plants’ height was recorded, and the height relative growth rate (HRGR) was computed as follows: HRGR = (height at d60—height at d0)/ (t60—t0).

The chlorophyll content, malondialdehyde (MDA) content, and the activity of antioxidant enzymes CAT, POD, and SOD were evaluated at the end of the Cd treatment. The total chlorophyll content was measured on two fully opened leaves with three technical measures per leaf, with a SPAD meter on the plants. In addition, the content of MDA and enzymatic activities of CAT, POD, and SOD were measured on leaf samples in triplicate using their specific kits purchased from Nanjing Jiancheng Bioengineering Institute, Nanjing, China [45].

### RNA isolation, cDNA library construction, de novo assembly, and DEGs analysis

Plant leaf samples were collected at the end of the Cd treatment in control (CK-d0) and treated plants (Cd-d0) in triplicate to explore the molecular mechanisms involved in Cd tolerance. This approach has been widely used in many species [11, 13, 29]. Additionally, Cd-treated plants were sampled ten days after the end of the treatments (Cd-d10) to examine changes in molecular mechanisms after exposition to Cd stress. All leaf samples were directly frozen in liquid nitrogen and stored at -80 °C until total RNA extraction. Leaf samples were preferred over roots for the transcriptome analysis as more DEGs were identified in leaves under Cd treatment compared to roots in mulberry [13].

Total RNA extraction from leaf samples, quality and integrity checking, sequencing on Illumina Hiseq 2000 platform, cDNA library construction, de novo assembly, and DEGs screening were carried out as per Chen et al. [12]. Briefly, the TRIzol kit (Invitrogen GA, USA) was used for total RNA extraction from samples following the instructions by the manufacturer. The quality, purity, and integrity were investigated using a NanoDrop ND-2000 spectrophotometer (Thermo Scientific, USA) at 260/280 nm. cDNA libraries were constructed from the leaf total RNA with the TruSeqTM RNA sample prep kit (Illumina, San Diego, USA) according to the manufacturer’s instructions. Next, the libraries were sequenced on the Illumina Hiseq X™ Ten platform by Shanghai Majorbio Biopharm Technology Corporation (Shanghai, China). Using the Seq-Prep program, the low-quality reads were discarded, and high-quality reads (clean reads) were generated. Finally, the clean reads were de novo assembled using the Trinity software [23, 24].

The expression levels of genes were assessed by FPKM (Fragments per Kilobase of transcript per Million mapped reads) using RSEM (www.biomedsearch.com/nih/RSEM-accurate-quantification-from/21816040.html) and Bowtie software (http://bowtie.cbcb.umd.edu). The DEGs were detected using DESeq software (https://www.rdocumentation.org/packages/DESeq2). The applied criteria for DEGs identification were threshold |log2Fold Change|≥ 1 and *p*-value < 0.5.

### DEGs analysis and functional annotation

BlastX (E-value > 10^–5^) against Swiss Prot, Protein family (Pfam) database, NCBI non-redundant (Nr) databases, Cluster of Orthologous Groups databases (COG/KOG), Kyoto Encyclopedia of Genes and Genomes pathway database (KEGG), Gene Ontology (GO), and Trembl was used for gene function annotation. To gain insights into the biological functions of DEGs, we conducted GO and KEGG pathway enrichment analyses through the Blast2GO [46] and KOBAS2.0 [47] programs, respectively.

### Quantitative real-time PCR (qRT-PCR) analysis

The qRT-PCR was achieved on LightCycler480 (Roche, Switzerland) real-time PCR system, with ChamQ™ SYBR1 qPCR Master Mix (Vazyme Biotech, Nanjing, China) as per Chen et al. [12]. In brief, approximately 2 μg of RNA from each sample was reverse-transcribed by reverse transcriptase M-MLV (TaKaRa). The histone Actin9 gene served as the internal control for transcripts normalization using the 2^−ΔΔCT^ method [48]. Three biological replicates were applied for each gene. The selected genes and their specific primers are listed in Table S1.

### Statistical analysis

Statistical analyses of all traits were conducted using R (www.r-project.org), and the data are presented as the mean ± SD of three replicates. Statistical differences were performed by t-test at *P* < 0.05. GraphPad Prism v9.0.0121 (GraphPad 159 Software Inc., La Jolla, CA, USA) was used to construct bar graphs and pies. The prcomp and pheatmap functions in R were used for PCA (principal component analysis) and HCA (hierarchical cluster analysis) analysis, respectively.

## Supplementary Information

**Additional file 1: Table S1.** List of primers used for the qRT-PCR analysis. **Table S2.** List of DEGs between CK-d0 and Cd-d0. **Table S3.** List of differentially expressed transcription factors (TFs) between CK-d0 and Cd-d0. **Table S4.** List of between CK-d0 and Cd-d0 related to main Cd homoestasis and detoxification mechanisms. **Table S5.** List of DEGs between Cd-d0 and Cd-d10.

**Additional file 2: Fig. S1.** Correlations analysis of samples. Control (CK-d0), stressed plants at the end of Cd treatment (Cd-d0) and ten days after the end (Cd-d10). **Fig. S2.** Distribution of transcripts and unigenes lengths. **Fig. S3.** Similarity rate of unigenes to known genes in other species. **Fig. S4.** HCA and PCA analysis of RNA-seq data of samples. Control (CK-d0), stressed plants at the end of Cd treatment (Cd-d0) and ten days after the end (Cd-d10). **Fig. S5.** Volcano plot of DEGs between CK-d0 and Cd-d0 (A), Ck-d0 and Cd-d10 (B), and Cd-d0 and Cd-d10 (C). **Fig. S6.** GO term enrichment results of the DEGs between CK-d0 and Cd-d0. **Fig. S7.** Heatmap of log2FC values of the DEGs enriched in heat shock proteins (A) and late embryogenesis abundant (LEA) proteins (B). **Fig. S8.** KEGG enrichment maps of the DEGs between CK-d0 and Cd-d0 involved in MAKP signaling pathway (A) and plant hormone signal transduction (B). **Fig. S9.** GO term enrichment results of the DEGs between Cd-d0 and Cd-d10. **Fig. S10.** KEGG annotation and enrichment results of the DEGs between Cd-d0 and Cd-d10.

## Data Availability

The raw transcriptome data has been submitted to NCBI SRA under the accession number: PRJNA851843 (https://www.ncbi.nlm.nih.gov/bioproject/?term=PRJNA851843). Other data generated or analyzed during this study are included in this published article and its supplementary information files.

## Abbreviations

ABA: Abscisic acid
TF: Transcription factor
qRT-PCR: Quantitative real-time PCR
PCA: Principal component analysis
HCA: Hierarchical cluster analysis
POD: Peroxidase
CAT: Catalase
SOD: Sodium oxide dismutase
HMA: Metal binding domain
KEGG: Kyoto Encyclopedia of Genes and Genomes pathway database
GO: Gene Ontology
MDA: Malondialdehyde
