# Supplementary material for: Physiological and transcriptional mechanisms associated with cadmium stress tolerance in Hibiscus syriacus L
Source: BMC Plant Biol. 2023 May 29;23:286. doi: 10.1186/s12870-023-04268-x (PMC10226262; doi:10.1186/s12870-023-04268-x)
Supplement: Supplementary file 2 — Additional file 2: Fig. S1. Correlations analysis of samples. Control (CK-d0), stressed plants at the end of Cd treatment (Cd-d0) and ten days after the end (Cd-d10). Fig. S2. Distribution of transcripts and unigenes lengths. Fig. S3. Similarity rate of unigenes to known genes in other species. Fig. S4. HCA and PCA analysis of RNA-seq data of samples. Control (CK-d0), stressed plants at the end of Cd treatment (Cd-d0) and ten days after the end (Cd-d10). Fig. S5. Volcano plot of DEGs between CK-d0 and Cd-d0 (A), Ck-d0 and Cd-d10 (B), and Cd-d0 and Cd-d10 (C). Fig. S6. GO term enrichment results of the DEGs between CK-d0 and Cd-d0. Fig. S7. Heatmap of log2FC values of the DEGs enriched in heat shock proteins (A) and late embryogenesis abundant (LEA) proteins (B). Fig. S8. KEGG enrichment maps of the DEGs between CK-d0 and Cd-d0 involved in MAKP signaling pathway (A) and plant hormone signal transduction (B). Fig. S9. GO term enrichment results of the DEGs between Cd-d0 and Cd-d10. Fig. S10. KEGG annotation and enrichment results of the DEGs between Cd-d0 and Cd-d10. [file 12870_2023_4268_MOESM2_ESM.pptx]

## Slide 1
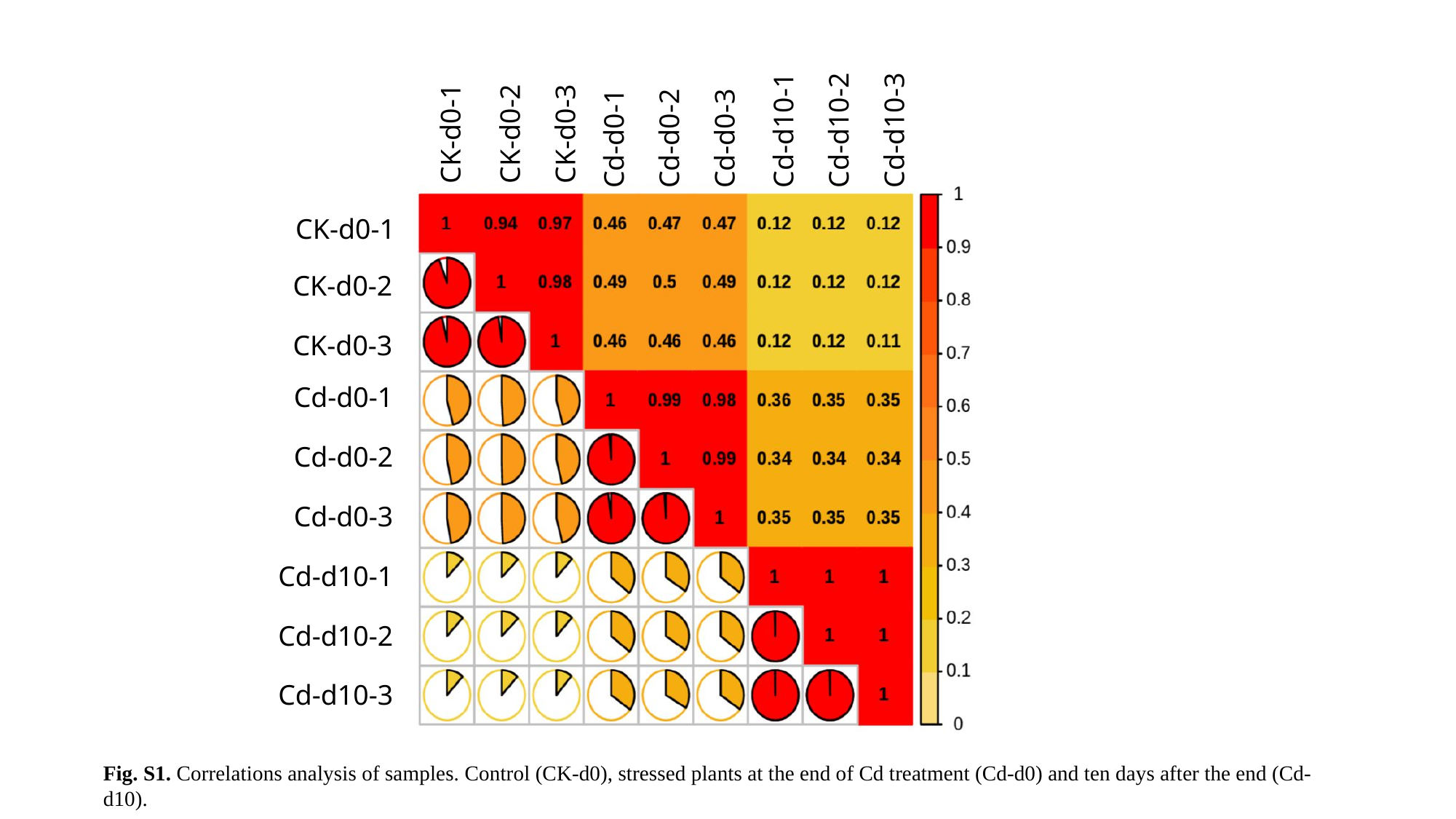

Cd-d10-1
Cd-d10-2
Cd-d10-3
CK-d0-1
CK-d0-3
CK-d0-2
Cd-d0-1
Cd-d0-2
Cd-d0-3
CK-d0-1
CK-d0-2
CK-d0-3
Cd-d0-1
Cd-d0-2
Cd-d0-3
Cd-d10-1
Cd-d10-2
Cd-d10-3
Fig. S1. Correlations analysis of samples. Control (CK-d0), stressed plants at the end of Cd treatment (Cd-d0) and ten days after the end (Cd-d10).

## Slide 2
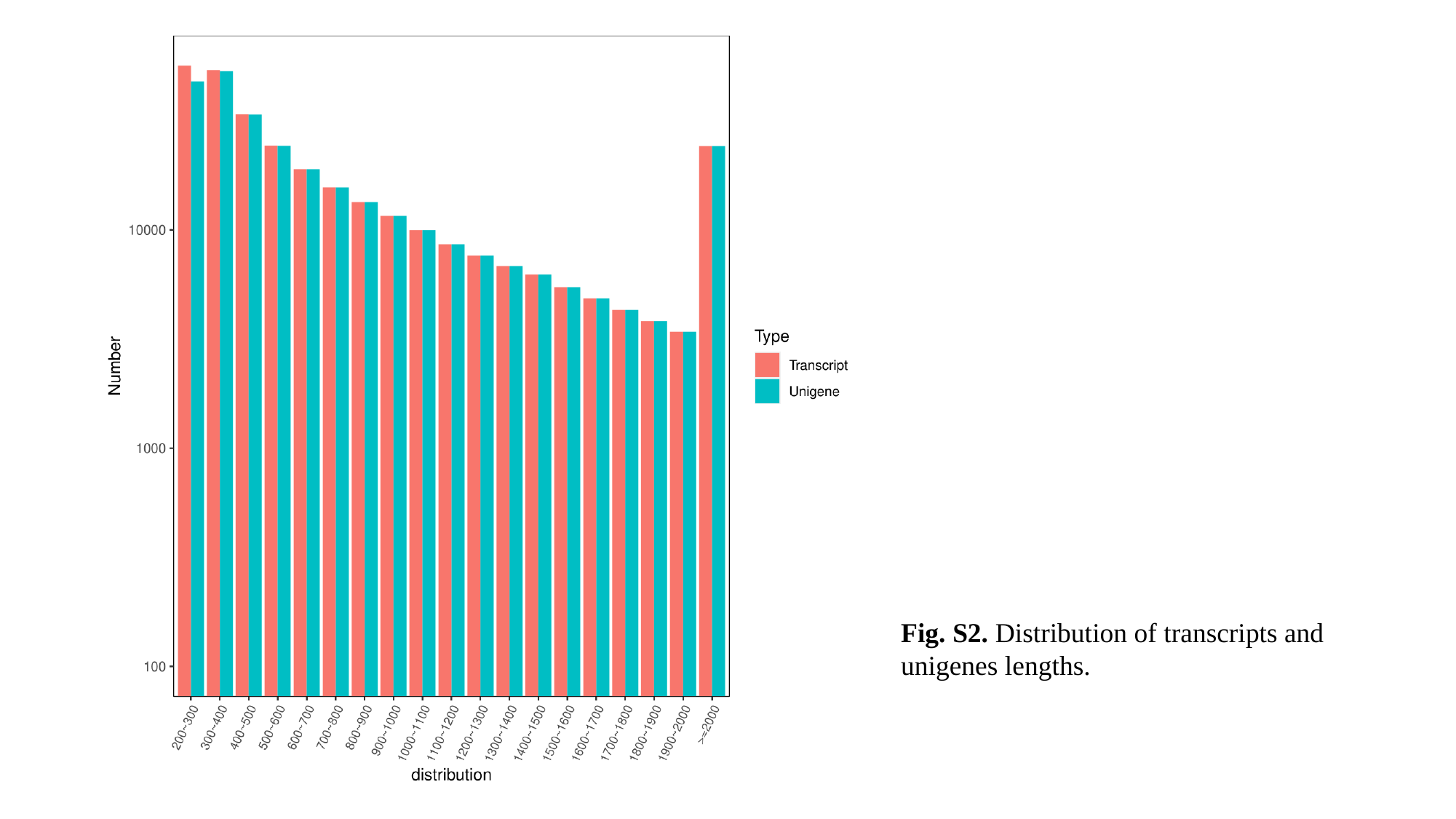

Fig. S2. Distribution of transcripts and unigenes lengths.

## Slide 3
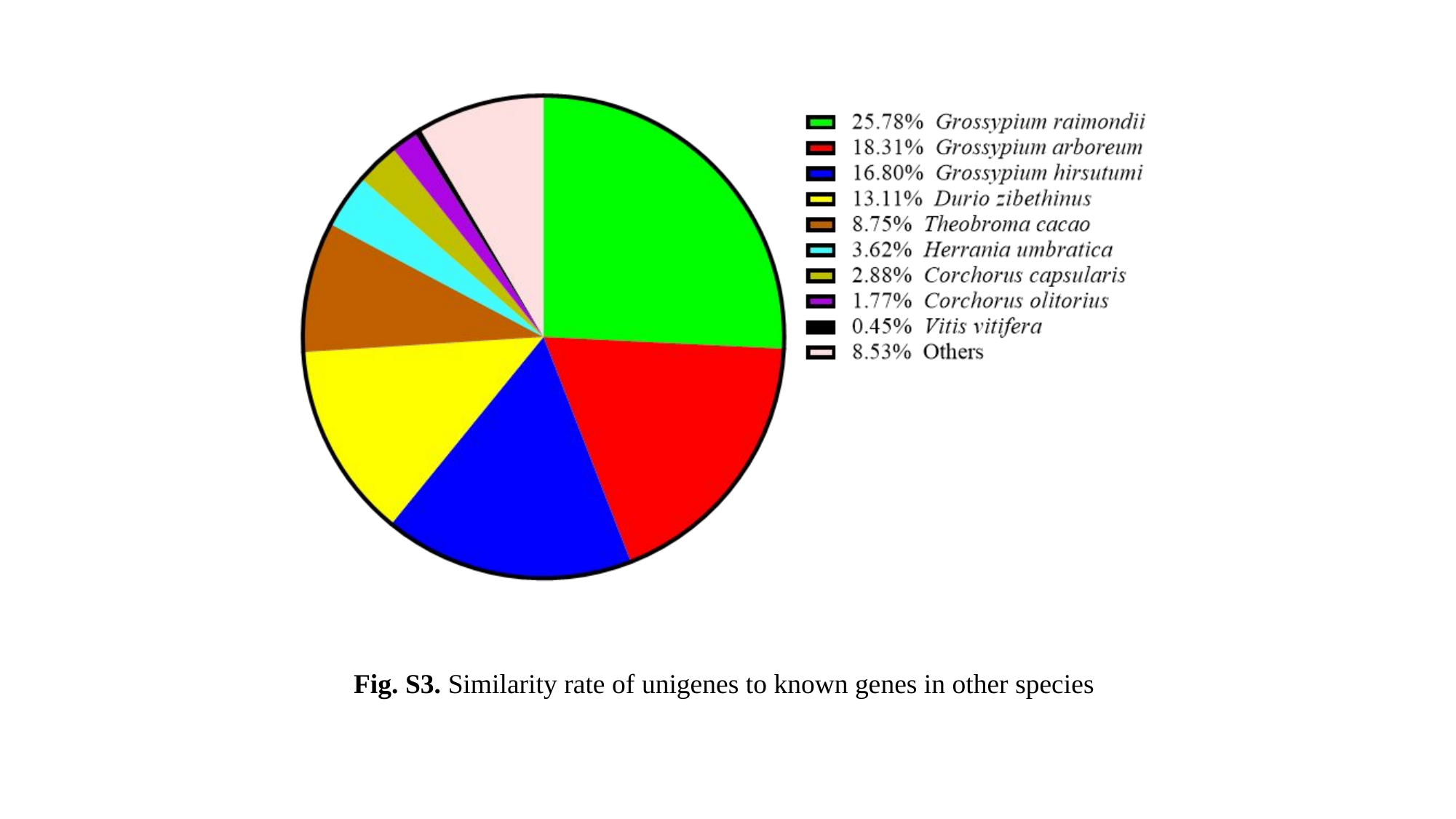

Fig. S3. Similarity rate of unigenes to known genes in other species

## Slide 4
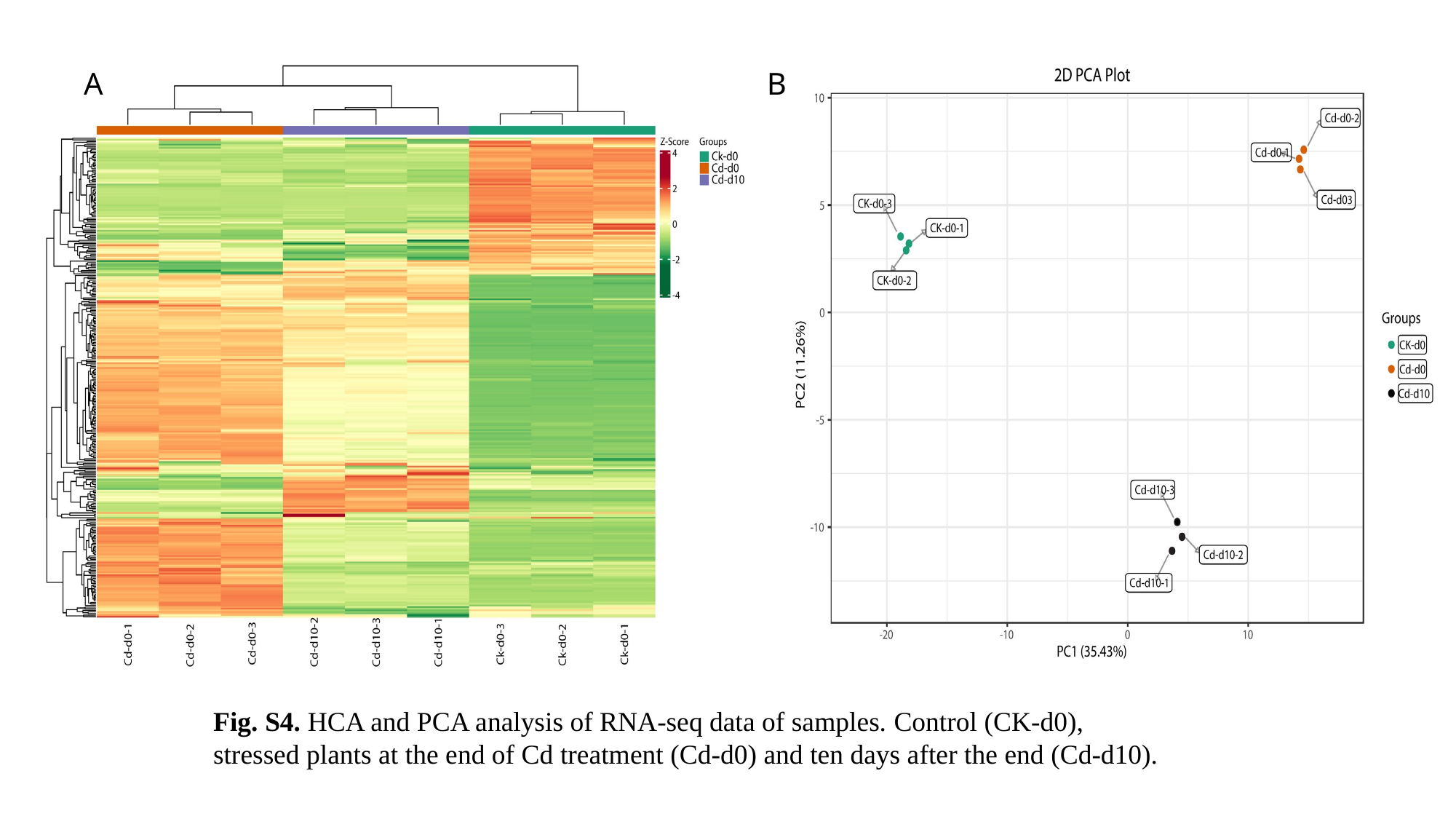

A
B
Fig. S4. HCA and PCA analysis of RNA-seq data of samples. Control (CK-d0), stressed plants at the end of Cd treatment (Cd-d0) and ten days after the end (Cd-d10).

## Slide 5
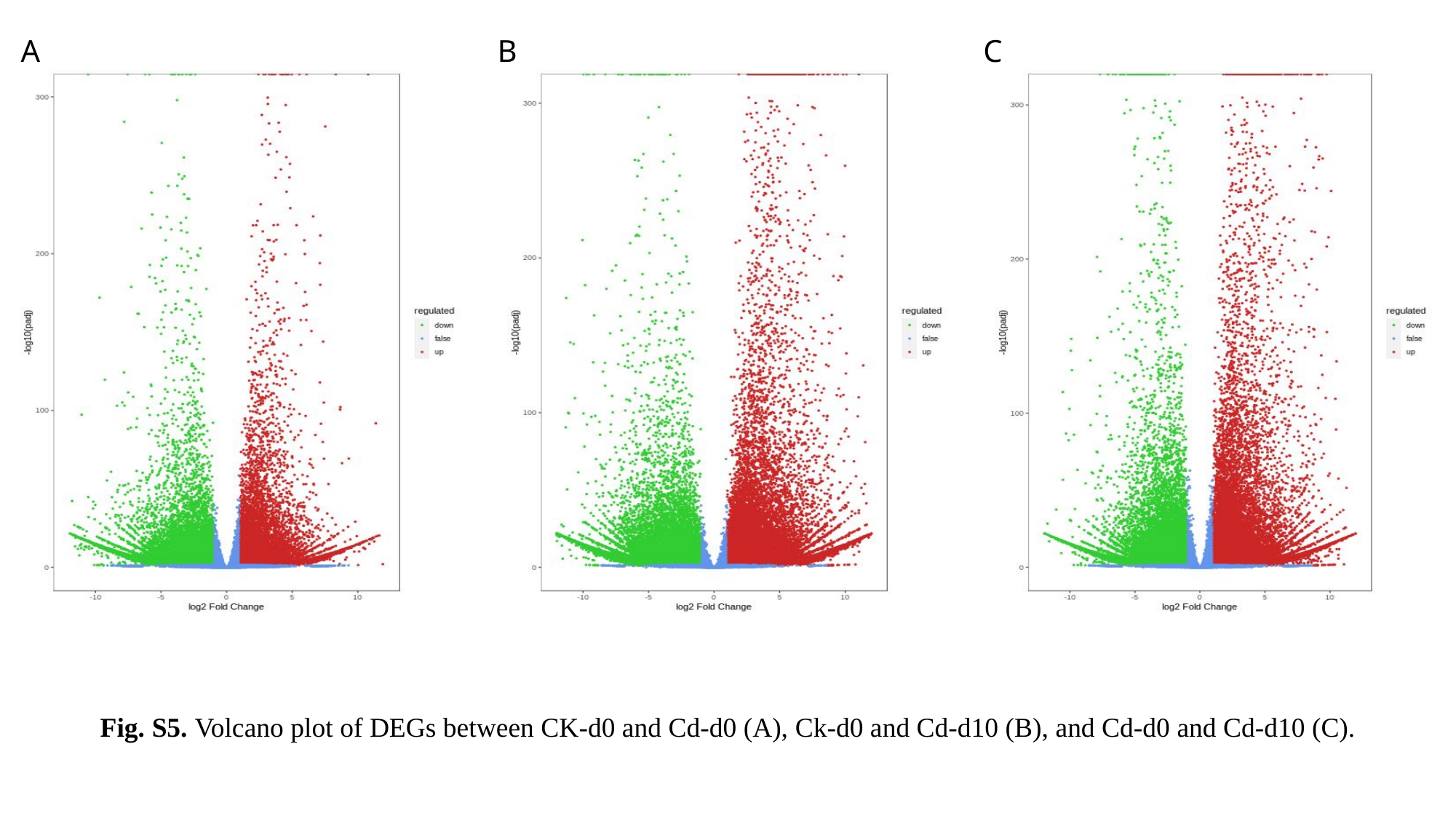

A
B
C
Fig. S5. Volcano plot of DEGs between CK-d0 and Cd-d0 (A), Ck-d0 and Cd-d10 (B), and Cd-d0 and Cd-d10 (C).

## Slide 6
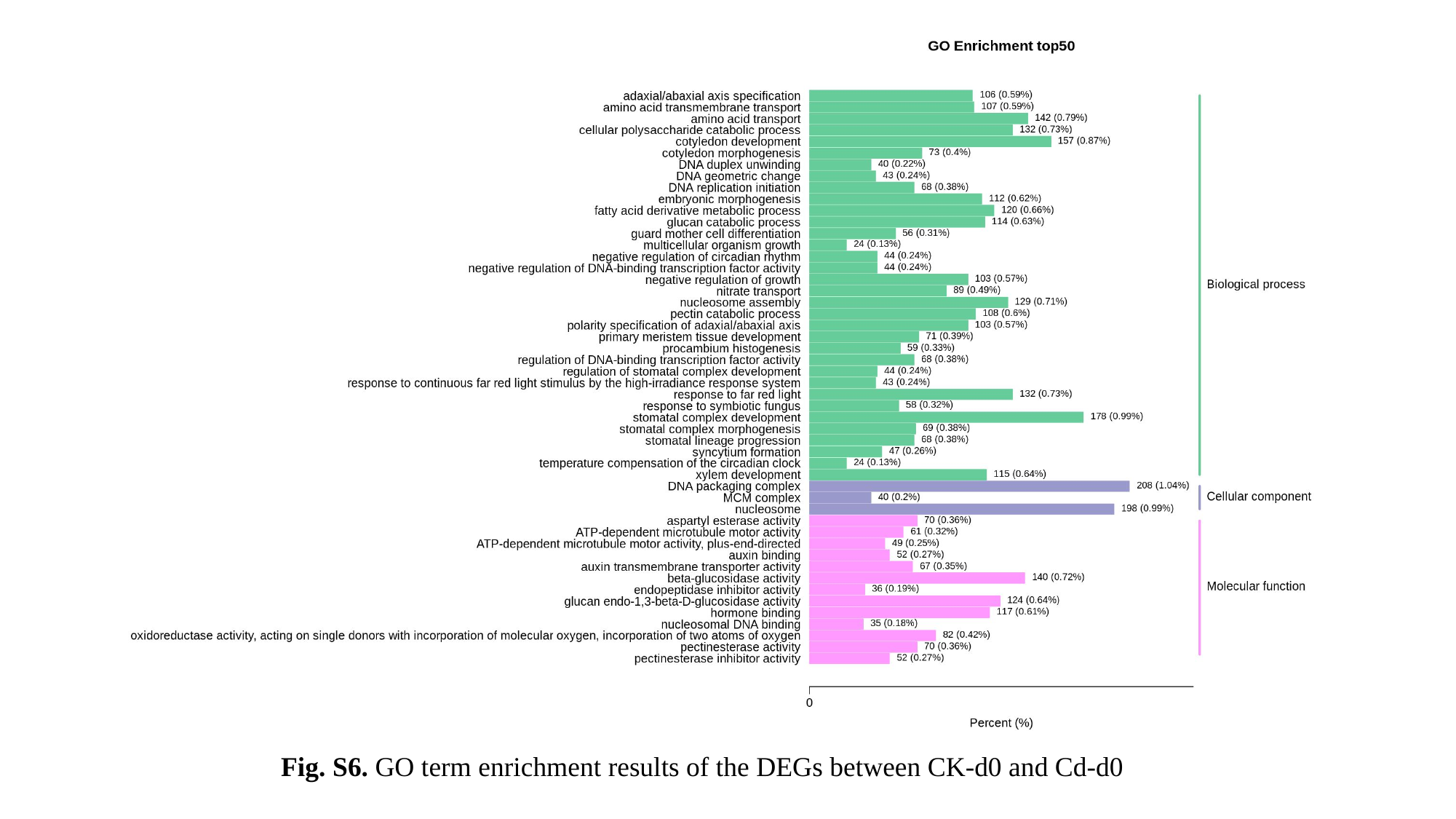

Fig. S6. GO term enrichment results of the DEGs between CK-d0 and Cd-d0

## Slide 7
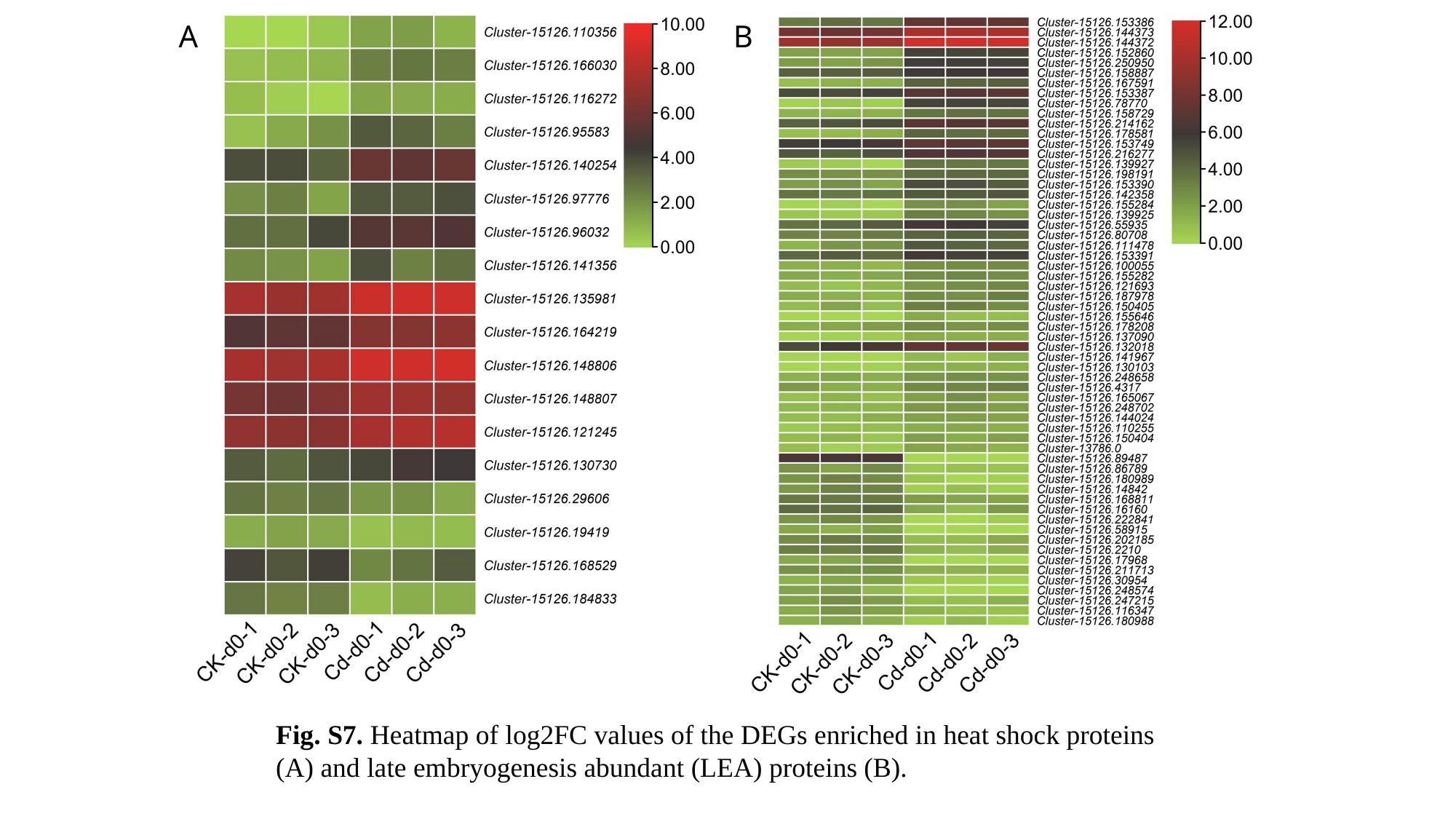

A
B
Fig. S7. Heatmap of log2FC values of the DEGs enriched in heat shock proteins (A) and late embryogenesis abundant (LEA) proteins (B).

## Slide 8
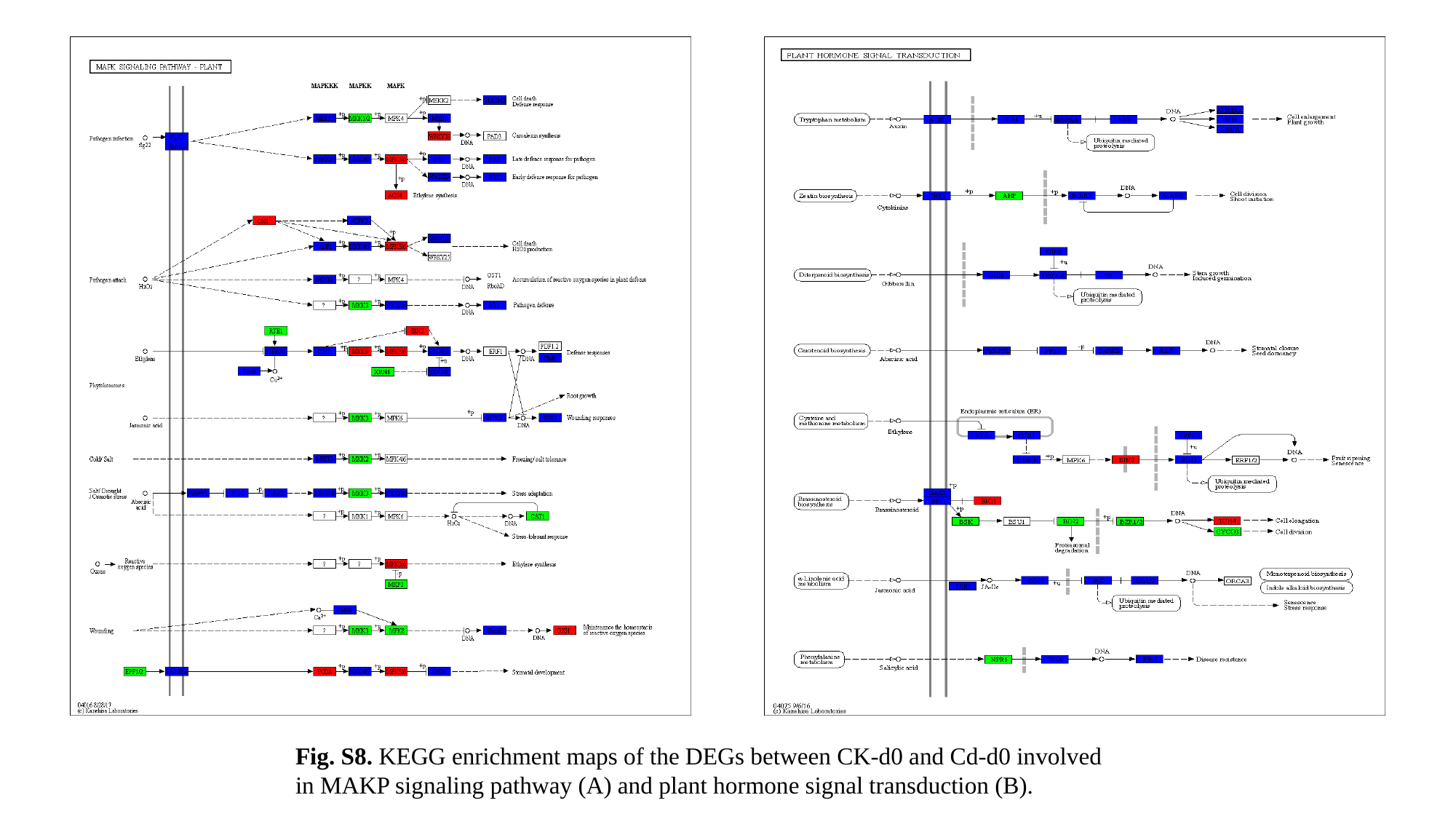

Fig. S8. KEGG enrichment maps of the DEGs between CK-d0 and Cd-d0 involved in MAKP signaling pathway (A) and plant hormone signal transduction (B).

## Slide 9
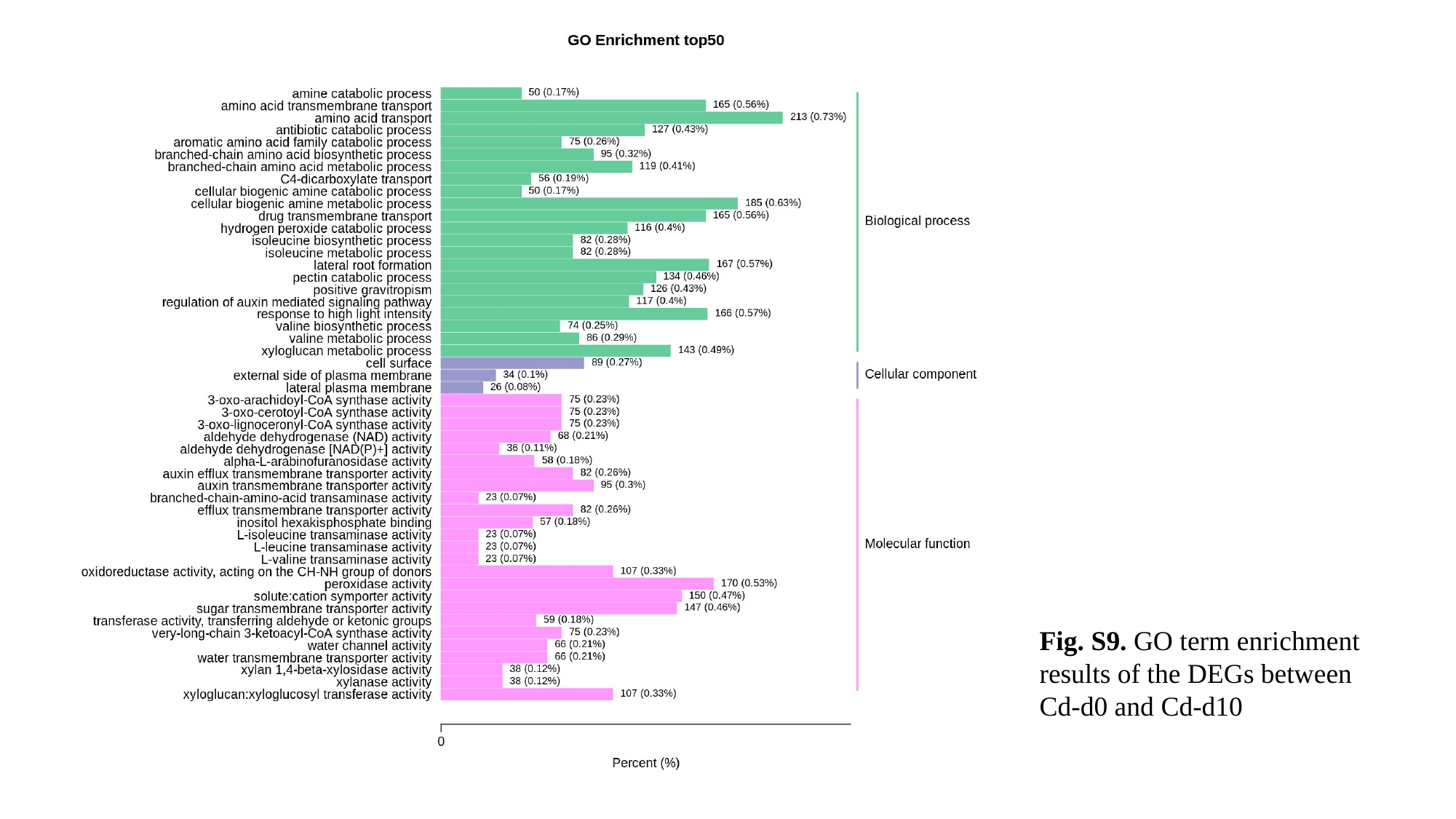

Fig. S9. GO term enrichment results of the DEGs between Cd-d0 and Cd-d10

## Slide 10
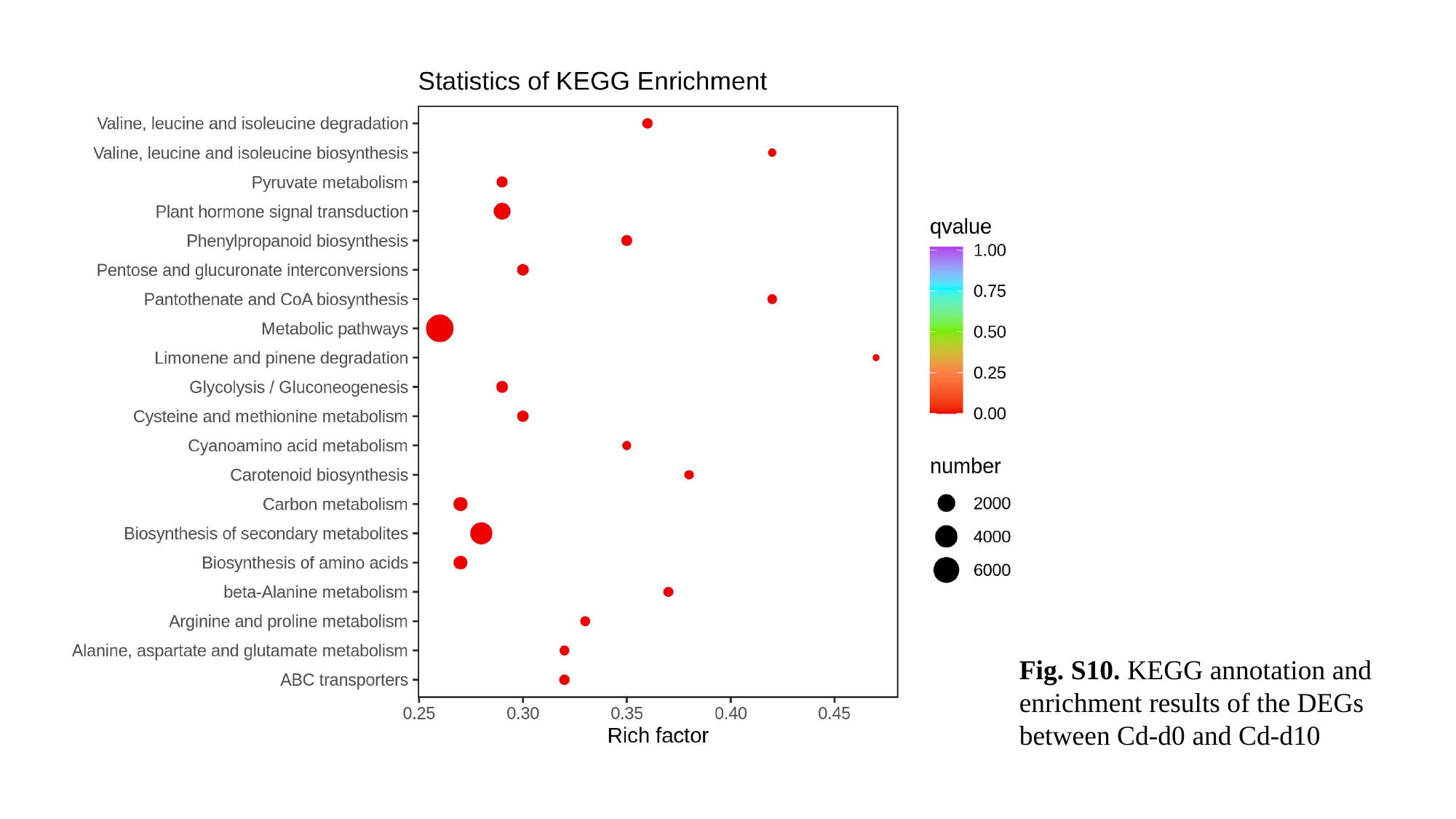

Fig. S10. KEGG annotation and enrichment results of the DEGs between Cd-d0 and Cd-d10
